# Concordance between different methods of detecting fungal-specific IgE antibody in asthma

**DOI:** 10.3389/falgy.2026.1788615

**Published:** 2026-04-01

**Authors:** Nadya Shifani, David W. Denning

**Affiliations:** Manchester Fungal Infection Group, Faculty of Biology, Medicine and Health, The University of Manchester, Manchester Academic Health Science Centre, Core Technology Facility, Manchester, United Kingdom

**Keywords:** commercial kits, concordance, fungal asthma, fungal sIgE, specific IgE

## Abstract

Documenting fungal sensitization plays a pivotal diagnostic role in asthma endotypes. Diagnosis relies heavily on detecting fungal-specific immunoglobulin E (sIgE), yet variability in allergen representation, cross-reactivity, and assay performance complicates standardization. Although ImmunoCAP is regarded as the reference method, many commercial kits are available worldwide, with varying accuracy. A scoping review was conducted using Arksey and O'Malley's five-stage framework. Literature searches in PubMed, Scopus, and ProQuest MEDLINE identified studies comparing fungal sIgE detection kits. Inclusion criteria required human studies evaluating fungal allergens in asthma-related diseases. Data from 16 eligible studies and 32 diagnostic kits were analyzed, focusing on assay characteristics, concordance, sensitivity, and specificity. There are 32 commercial kits available, which vary in detection principles, including enzyme immunoassays, immunoblots, and microarrays, with positivity thresholds ranging from 0.1 to 0.35 kUA/L. Most assays identified *Aspergillus* and *Alternaria-*specific IgE, while fewer detected *Cladosporium*, *Candida*, or *Trichophyton*. Concordance with ImmunoCAP ranged from moderate to substantial, with the strongest agreement reported for *Alternaria*. ImmunoCAP demonstrated the highest sensitivity and specificity; however, emerging systems, such as the GOLD chip and MeDALL microarray, showed promising improvements in multiplex capacity and detection limits. Meanwhile, several commercial assays have no publications documenting their diagnostic performance. Fungal sIgE detection remains heterogeneous. Newer microarray-based assays offer enhanced sensitivity and broader allergen profiling. Standardizing cutoff thresholds and incorporating recombinant fungal allergens are crucial for strengthening diagnostic precision and improving the clinical management of fungal asthma.

## 1 Introduction

Immunoglobulin E (IgE) is the least abundant immunoglobulin isotype, but measuring its serum levels is crucial for diagnosing allergic conditions (1), parasitic infections (2), and other related diseases, including some autoimmune (3) and chronic diseases (4). Specific IgE (sIgE) assays are recommended to confirm sensitization in IgE-mediated diseases, especially in cases of food and respiratory allergies (5). The detection of allergen-specific IgE antibodies can be conducted either *in vivo* (skin prick tests) or *in vitro* (6) (7),. Component-resolved diagnostics (CRD) using individual protein antigens in specific IgE assays have also revolutionized the diagnosis of allergies (8). This refinement is particularly relevant as several fungal genera have been implicated in asthma pathogenesis through both IgE and non-IgE-mediated mechanisms. Among these genera*, Aspergillus fumigatus, Alternaria* spp., and *Cladosporium* spp. are recognized as the most common causes of allergies and sensitization, including a localized form of IgE-mediated pathology known as allergic fungal rhinosinusitis (AFRS) (9–11).

AFRS accounts for approximately 6%–9% of cases of chronic rhinosinusitis requiring surgical intervention, with a higher prevalence in warm/humid climates (12). Alongside AFRS, sensitization to fungi, such as *Aspergillus*, is common and is a significant factor in allergic bronchopulmonary aspergillosis (ABPA), a condition often associated with asthma or with cystic fibrosis (13–15). ABPA affects an estimated 1%–2% of adult asthma patients globally, rising to 10%–15% in severe asthma and tertiary referral cohorts (13, 16). Many other fungi cause allergic bronchopulmonary mycosis (ABPM) (17, 18). ABPM accounts for a smaller proportion with a prevalence below 1%, and its occurrence varies geographically. Additionally, ABPM caused by fungi other than *Aspergillus* is much less common, with *C. albicans* being the most frequent cause, accounting for 60% of cases (17). In contrast, fungal sensitization without manifestations of ABPA/ABPM, such as central bronchiectasis or hyperattenuating mucus, is termed severe asthma with fungal sensitization (SAFS). Sensitization to *A. fumigatus*, a significant contributor to SAFS, is found in 16% of adults and up to 20% of children with severe or treatment-resistant asthma (19). Collectively, ABPA, ABPM, and SAFS are referred to as ‘fungal asthma’, affecting approximately 10%–20% of all asthma cases (19–21).

The link between AFRS, ABPA, and asthma is primarily mediated through shared immunopathological characteristics, including elevated total and specific IgE levels (22). About 80% of ABPA patients have co-existing AFRS, and around 70% of those with severe asthma are sensitized to one or more of about 80 different fungi (23). Research on the link between fungal sensitization and asthma is complicated by the lack of standardized methods to measure fungal-specific IgE levels. Fungi produce numerous allergens; for example, *A. fumigatus* has more than 60 IgE-binding proteins. Additionally, there is an overlap in individual protein antigens across different fungal species (24). This diversity and complexity challenge the production of standardized preparations by manufacturers and the definition of reference units, resulting in variable reproducibility and inconsistent IgE testing across platforms, particularly in commercial kits (25). Among all commercial kits reported so far, ImmunoCAP is recognized as the “reference standard” for *in vitro* IgE testing, supported by over 4,000 scientific articles that demonstrate its clinical value (26). However, in the absence of component-resolved diagnostics, ImmunoCAP falls short in accurately differentiating between true primary sensitization and cross-reactive responses (27). Several countries have developed their specific IgE detection assays, such as the Oriton IgE kit in Japan (28), Ouboke in China (29), and PROTIA Allergy-Q in South Korea (30). In addition, the emergence of allergen microarray technology has led to initiatives like the European Union-funded project MeDALL (31). This paper reviews the effectiveness and performance of commercial kits for detecting fungal-specific IgE.

## 2 Literature selection

We conducted a scoping review using the five-stage framework proposed by Arksey and O'Malley (32). Ethical approval was not required. An extensive literature search was conducted utilizing the databases Scopus, PubMed, and ProQuest MEDLINE. The search employed specific keywords (i.e., fungal sIgE, detection kits, and fungal asthma), with no restrictions on publication date to ensure all relevant studies were included; the searches were conducted in July 2025. Inclusion and exclusion criteria were established to select relevant studies. The inclusion criteria required that papers must (1) include a suitable comparison of IgE detection kits; (2) include targeted populations of children and adults; and (3) include fungal allergens in diseases associated with asthma. The exclusion criteria were (1) lack of full text availability; (2) books, letters, poster/conference abstracts, case reports, reviews, meta-analyses, or conference papers; (3) animal studies; (4) follow-up studies, (5) non-related diseases to asthma, and (6) undefined or nonspecific fungal allergens.

All studies from each database were exported into EndNote 20 for management and selection. The characteristics of 32 specific IgE diagnostic kits from various countries are summarized descriptively, including their cutoff values and measurement principles. The studies were organized and reviewed based on performance comparisons of diagnostic assays, including concordance, sensitivity, and specificity. No meta-analysis was conducted due to the distinct nature of the studies, which varied in comparison metrics, allergen types, and subject and control groups.

## 3 Results

The database searches yielded a total of 863 articles, of which 294 were discarded beforehand as they were classified as ineligible by automated tools, leaving 569 for review. After screening the titles and abstracts, 441 articles were excluded, leaving 128 papers selected for comprehensive evaluation. Upon conducting the full-text review, 112 articles were excluded, resulting in 16 studies that provided original data using sIgE detection kits for fungal allergens associated with asthma. In addition to a systematic search of electronic databases, supplementary data were extracted from the official websites and instructions for use (IFU) documents of commercially available specific IgE (sIgE) detection kits to capture detailed information regarding the technical specifications and cutoff characteristics of each assay, often not described in peer-reviewed literature.

Supplementary Table S1 shows that 32 commercial sIgE detection kits from 13 different countries are available, with distinct analyte coverage for various fungal allergens. These commercial assays employ various detection principles, primarily enzyme immunoassays (EIA). A positivity threshold widely used across assays is ≥0.35 kU/L, ranging from as low as 0.1 kUA/L (IDS sIgE, UK) to semi-quantitative formats that lacked fixed cutoff values. Almost all kits (30/32) were able to identify *Aspergillus* and *Alternaria* species-specific IgE, while *Penicillium* and *Candida* had a slightly lower representation (20/32). About half of the kits could also detect *Cladosporium* species-specific IgE (16/32). Conversely, *Trichophyton* was the least commonly included allergen (7/32). ImmunoCAP, ALEX-2, and EUROLINE provided extensive coverage of all six fungal genera. Significantly, regional kits such as the Oriton IgE (Japan) and PROTIA Allergy-Q (South Korea) also demonstrated solid allergen representation, with regionally appropriate validation parameters. Out of the 32 kits listed in this study, two kits are not commercially available because they are considered research-grade assays. Those are the MeDALL chip, part of the EU-funded project, and the GOLD chip project from South Korea. Most kits rely on the crude/natural fungal antigens for IgE detection, except ImmunoCAP ISAC, ALEX-2, DELFIA, and Gold-chip. These four kits offer component-resolved diagnosis (CRD), which uses purified recombinant proteins to measure IgE against individual molecular components rather than the whole-extract mixtures.

The inter-assay concordance among various allergens and commercial kits is summarized in Supplementary Table S2. This analysis includes thirteen studies published between 2011 and 2024. The studies used metrics such as intraclass correlation coefficients (ICCs), Cohen's kappa (κ), and overall agreement percentages to evaluate performance. Most studies reported moderate to substantial concordance (κ values ranging from 0.528 to 0.923) between the newer systems and the ImmunoCAP gold standard. For instance, an agreement of up to 96% for *Alternaria* detection between Immulite 2000 and ImmunoCAP (28), while up to 96.63% concordance was observed using PROTIA Allergy-Q (31). In contrast, the findings of Yoshikawa et al. found that fungal allergens, such as *Candida*, showed lower correlation values in some cases (32). These variations suggest the need for kit-specific calibration, particularly for fungi with lower prevalence or higher antigenic variability.

Furthermore, a more detailed analysis of specific fungal allergens is presented in Supplementary Table S3. Three studies focused exclusively on fungal-specific IgE assays. Kespohl et al. (33) found that skin prick tests (SPTs) identified more mould sensitisations (90/168) than ImmunoCAP (56/168), though the concordance varied significantly across fungal species. Kuwabara et al. (34) reported a 98.2% concordance between ImmunoCAP and View Allergy for the detection of *A. fumigatus*, whereas MAST IV showed reduced performance (77.2%). Huang et al. (35) assessed four kits (ImmunoCAP, BioIC, EUROLine, and Ouboke) in patients with ABPA. ImmunoCAP demonstrated the highest sensitivity and specificity (88.9% and 100%, respectively) for *A. fumigatus.* In contrast, BioIC and EUROLine showed sensitivities below 45%, emphasizing significant variability among kits in fungal-specific IgE detection. Collectively, these results indicate that while several commercial kits perform adequately in detecting fungal-specific IgE, ImmunoCAP remains the most validated and reliable across diverse populations. Nonetheless, emerging advanced development assays, such as the GOLD chip, demonstrated excellent performance, with a limit of detection lower than 0.35 IU/mL and strong agreement (∼92%) with ImmunoCAP results. Its microarray format also enables parallel, simultaneous testing of multiple allergen extracts while requiring only a small sample volume (less than 30 µL), thereby improving accuracy (36).

## 4 Discussion

### 4.1 Specific-IgE detection assay formats

In the historical timeline of specific IgE detection, the RAST (Radioallergosorbent Test) method is the oldest, first published in 1967 (33). The early RAST assays tested over 20 allergens and demonstrated correlation with clinical challenge testing (34). It was a non-competitive, immuno-radiometric assay that employed allergens attached to allergo-sorbent paper discs. From a clinical point of view, this system has been rarely used as it was incapable of detecting low-affinity IgE (35). Since 2010, NIAID/NIH has advised against using RAST for allergies, recommending more sensitive and safer tests instead (6). Despite this, many clinicians still colloquially refer to modern blood IgE assays as “RAST,” even though they are fundamentally different. The IgE Antibody REAST kit (HOB Biotech Group) is an example. Although REAST and RAST may sound similar, these two methods differ in assay format, labeling methods, and the capture and detection of IgE. In REAST, anti-IgE antibodies are first coated, making it safer because it does not use radioactive labels. Additionally, REAST often has higher sensitivity and specificity compared to traditional RAST (36, 37).

Seeking non-radioactive alternatives for RAST, researchers developed enzyme-linked immunoassays (EIAs/ELISAs) beginning in the late 1960s (38, 39). EIAs offered safer, scalable detection of specific IgE without radioactivity, and rapidly became the standard in later decades. The most common detection modalities under EIA include colorimetric, fluorescent, and chemiluminescent readouts. The colorimetric detection relies on a colour change, while FEIA and CLEIA use enzymatic reactions, producing fluorescent signals and emitting light, respectively (40). In terms of sensitivity, colorimetric readouts have the lowest sensitivity, while chemiluminescence offers the highest sensitivity (41). Of the 32 commercially available kits, 22 use enzyme-linked immunoassay, representing over 65% of the total. However, only half of these kits (11 out of 22) have had their concordance and performance externally assessed through publication, leaving the performance of the other half uncertain. The 11 externally assessed kits include ImmunoCAP, OPTIGEN, AdvanSure, HYTEC 288, IMMULITE 2000, BioIc, Oriton, AlaSTAT, View Allergy, and Ouboke. The NOVEOS systems, which also utilize an immunoassay format, demonstrated a good agreement with ImmunoCAP for detecting food and airborne allergies (42). However, to date, no peer-reviewed study has demonstrated its capacity to identify fungal allergens; only internal company documentation detailing its performance in detecting *Aspergillus* and *Alternaria* has been reported (43).

The immunoblot formats (including line blot) emerged as alternatives around the 1990s–2000s (44). Immunoblot assays are semi-quantitative and allow simultaneous detection of allergen proteins. Among the diagnostic kits identified in this study, EUROLINE stands out as a highly regarded immunoblot-based kit. Its performance demonstrates significant concordance with ImmunoCAP (45), and its specificity for fungal allergen mixtures reaches 100% (29). The other well-reviewed immunoblot kits are RIDA qLine and Polycheck Allergy. However, compared with the ImmunoCAP system, considered the gold standard, the concordance between these two kits is lower than that of EUROLINE (Supplementary Table S2).

Since the 2000s, allergen sIgE microarray assays have been developed. These assays are high-throughput multiplex systems detecting IgE to dozens to hundreds of allergen components simultaneously. The potential value of microarrays in allergic sensitization was first documented in 2002 (46), and it was accompanied by numerous follow-up studies utilising the same array, commonly referred to as the ISAC (Immuno Solid-phase Allergen Chip) system (6). The initial version of the ISAC microarray featured 74 allergenic proteins (ISAC74). Currently, the number of allergens on the microarray has increased, and a newer version containing 112 allergens (ISAC112) is available. Microarray technology represents the latest advancement regarded as an effective, multi-allergen method for profiling specific IgE levels using a minimal amount of serum sample (47). Among the four microarray kits used in this study, the MeDALL (Mechanisms of the Development of Allergy) developed by the MeDALL European project stands out as a novel technology. It features 170 allergens, surpassing the ISAC 112 kit (31, 48). The MeDALL chip offers new insights into the concept of the “allergen march,” which assesses allergic sensitization from childhood through adolescence (49). Furthermore, some studies indicate that it is more effective at detecting allergic sensitization compared to ImmunoCAP sIgE or skin prick tests (6, 48). To evaluate the performance of these diagnostic kits, several statistical measures can be applied. The sensitivity metric shows the proportion of true positives, while specificity indicates the proportion of true negatives. Values above 90% are generally considered excellent (50, 51).

Potential competition between allergen-specific IgG and IgE has been described in allergen microarrays, particularly when high IgG concentrations are present. This susceptibility arises because microarray tests use much lower levels of allergens on the solid phase than singleplex tests. As a result, this can impair the quantitative accuracy of the assays and may bias the analysis toward high-affinity IgE antibodies (52). To address this issue, some microarray designs have been modified to reduce IgE-IgG competition. For example, increasing the allergen spot density enhances IgE-binding capacity, ensuring that IgG-blocking antibodies become saturated and cannot outcompete IgE (53, 54). Some microarray platforms, such as ImmunoCAP ISAC, also have dual-fluorescence readout and calibration controls to achieve more accurate IgE quantification even in the presence of competing IgG antibodies (27). Another study also found that microarrays with silicon elements exhibit greater sensitivity for IgE-IgG reactivity to inhalant allergens (55). While this phenomenon of IgE-IgG competition in microarray is well documented for food and some inhalant allergens, data specific to fungal allergens remain sparse. The available literature on fungal allergen microarrays does not specifically address or discuss IgE-IgG competitive binding as a technical concern. This represents a potentially underexplored area in fungal allergy diagnostics, particularly given the importance of accurate IgE quantification for treatment decisions. Therefore, further research specifically examining IgE-IgG competition during microarray testing of fungal allergens would be valuable.

### 4.2 Cost and performance analysis

Some kits in this study are available globally, while others are region-specific. The most widely used sIgE testing kit is ImmunoCAP, offering over 550 specific allergen tests internationally (56). Among the current ImmunoCAP variations, the ISAC version, which uses microarray technology, has the highest system cost. A cost analysis indicated that a typical ImmunoCAP microarray requires a LuxScan reader, which incurs a capital expense of £28,999, plus an approximate kit price of £2,500. Additionally, personnel costs for performing and interpreting the tests range from £125 to £154 per kit per patient (57). Various global allergy guideline panels highlight that ImmunoCAP has replaced RAST in more than 80% of clinical laboratories (58). Studies also consistently show excellent analytical sensitivity (LoQ ∼0.1 kUA/L), with the typical positivity cutoff set at ≥0.35 kUA/L (59, 60). In addition to the ImmunoCAP, other internationally available diagnostic kits mainly originate from Europe. These include ALEX-2, DELFIA, EUROLINE and IMMULITE 2000. These kits are offered in UK panels, with prices ranging from approximately £200 to £600, depending on the number of allergens tested. On the other hand, the PROTIA Allergy-Q is a significant global commercial kit originating from an Asian country. It is distributed in over 70 countries and is featured at international trade exhibitions. In terms of price, it falls within the mid-range, with the PROTIA-Q44 (43 allergens) kit costing approximately £360–380 and the PROTIA-Q96 (107 allergens) kit costing approximately £425–450. The more affordable option is kits using traditional ELISA, such as the BioLINE and Ouboke, which cost about £20–40 per allergen for basic domestic use. Although these kits appear to be more affordable, their availability is likely limited to specific countries or regions.

### 4.3 Clinical relevance of fungal sensitization in asthma

Fungi are found worldwide, with more than 100,000 species identified. Only a few hundred are opportunistic pathogens, and approximately 80 genera are known to cause type I allergies, such as allergic rhinitis, asthma, and atopic dermatitis. The main allergenic fungi are from the phylum Ascomycota, followed by Basidiomycota and Zygomycota. The genera *Alternaria, Cladosporium*, *Aspergillus,* and *Penicillium,* all from Ascomycota. Other fungi that are less significant in terms of allergens include *Fusarium,* and *Curvularia* (Ascomycota), *Malassezia* (Basidiomycota), or *Rhizopus* (Zygomycota) (61). Many fungal extracts used in allergy diagnostics still carry taxonomic names that have since been updated or replaced. These names persist in clinical panels and labeling due to historical practice and regulatory consistency. For instance, *Cladosporium herbarum* was previously known as *Byssus herbarum, Dematium herbarum*, and *Heterosporium epimyces.* Despite reclassification, the older names remain in clinical use due to their continued use in extract manufacturing and databases (62). *Ulocladium botrytis,* was formerly grouped under *Alternaria* species due to morphological similarities. Even *Ulocladium* is now recognized as a distinct genus, patients may still be exposed to its allergen and be detected as *Alternaria* in some panels (63).

Most fungal sIgE detection kits typically use natural/crude extracts or fungal mixtures. However, studies have shown that using recombinant proteins can enhance specificity, avoid cross-reactivity, enable molecular-level sensitization profiling, and improve detection compared to extracts (64, 65). Natural extracts may cause false-positive IgE reactions due to carbohydrate structures (anti-CCD IgE), while recombinant proteins do not (66). Among commonly used recombinants, a study in Japan (67) revealed that rAsp f1 and rAsp f2 of *A. fumigatus* are particularly effective in diagnosing ABPA. Some studies also reported that up to 98% of *A. alternata* sensitizations can be detected only with rAlt a 1 (68), and over 80% of patients with ABPA exhibit sensitization to rAsp f1 (69). However, the 2024 ISHAM-ABPA guideline (12) highlighted that recombinant-based IgE testing is recommended as an adjunct to the core diagnostic criteria. The panel emphasizes that the total IgE is the main laboratory marker for assessing therapeutic response, rather than specific IgE. Changes in *Aspergillus*-specific IgE do not reliably indicate disease activity and should not guide therapy adjustments since they can remain elevated after clinical remission. In contrast, total IgE reflects the overall Th2-driven inflammatory load and responds more predictably to effective treatment.

The 0.35 kUA/L cutoff value is the most common threshold for fungal sIgE sensitization, with 21 out of 32 diagnostic kits reviewed in this study utilizing it (Supplementary Table S1). However, some studies evaluate cutoffs of 0.35, 0.5, and 1.0 kUA/L for *A. fumigatus* and recombinant components like rAsp f1/2 found that a higher threshold (0.70 kUA/L) better differentiated ABPA from simple sensitization among asthma patients (70). Another study argues that a single, arbitrarily low cut-off for all rAsp antigens is likely to result in poor diagnostic performance. Fixed cut-offs (0.35, 0.5, 1.0 kUA/L) performed poorly for ABPA because many *Aspergillus*-sensitized asthma (ASA) patients also exceed these low thresholds, yielding low specificity (often <70%). By maximizing the Youden index (sensitivity + specificity – 1), they found that the key cut-offs are 4.465 kUA/L for rAsp f1 (sensitivity ≈ 89%, specificity ≈ 100%) and 1.300 kUA/L for rAsp f2 (sensitivity ≈ 100%, specificity ≈ 81%). These higher values markedly improve specificity and sensitivity, thereby reducing false-positive diagnoses and misclassification between ABPA and ASA (69).

In most commercial fungal-specific IgE panels, *A. fumigatus* and *A. alternata* are consistently included (Supplementary Table S1). This prioritization is based on epidemiological prevalence, and strong clinical associations with severe asthma endotypes (Supplementary Table S4). *A. fumigatus* plays a role in asthma through a process that starts with the continuous inhalation of its small (2–3 µm) conidia, which easily penetrate the lower airways and remain present in moist indoor or work environments. In susceptible individuals, the fungus attaches to epithelial cells, germinates, and releases allergenic hyphal fragments. *A. fumigatus* has 72 identified allergens out of 195 found in *Aspergillus* spp (42). These allergens bind to IgE on mast cells and basophils, triggering a classic type I hypersensitivity reaction and downstream Th2 cytokines that amplify eosinophilic inflammation. The resulting airway inflammation promotes mucus hypersecretion and structural changes, including bronchial wall thickening and bronchiectasis. Repeated fungal impaction and chronic colonization further stimulate IgE-mediated immune responses, leading to ABPA or SAFS, both characterized by poor asthma control, frequent exacerbations, and reduced lung function (19).

*A. alternata,* with 309 allergenic molecules identified, is another of the most prevalent fungi sensitizers in the SAFS phenotype (71). Recognition of SAFS may prompt consideration of targeted interventions, including antifungal therapy, reducing environmental exposure, optimizing inhaled corticosteroid doses, and evaluating biological treatments. Randomized controlled trials and observational studies have shown that systemic azole antifungal treatments can enhance asthma control, quality of life, lung function, and reduce exacerbation frequency in selected SAFS patients (72). Similarly, another study suggested that voriconazole and posaconazole could be effective alternative options for treating SAFS and ABPA (73). Furthermore, *A. alternata* species is also often associated with thunderstorm asthma, a condition characterized by asthma exacerbations that occur during thunderstorms, particularly during pollen and mold seasons (74). Major allergen Alt a 1 is highly immunogenic, eliciting strong IgE responses and promoting airway inflammation by inducing epithelial damage and releasing Th2 cytokines. Although Alt a 1 is the major allergen of *A. alternata*, other allergens, such as Alt a 6 and Alt a 14, should also be included in the fungal allergy diagnosis panel. Unlike many fungi, where higher thresholds are required, even low-level IgE positivity to *Alternaria* (≥0.35 kUA/L) has been linked to an increased risk of exacerbation (75).

Aside from *A. fumigatus* and *A. alternata*, which are the primary fungi studied in asthma research, other fungi have also been linked to asthma development. *C. herbarum* is one of the most researched fungal species in allergy studies, following these two. Sensitization to *Cladosporium* is linked to increased asthma severity in several epidemiologic studies and has been linked to the surge of emergency asthma admissions in temperate climates (76). Levels of 3000 *Cladosporium* spores per cubic meter of air are usually considered the threshold for clinical significance. Cla h 8, a mannitol dehydrogenase, was recognized to be the major allergen of *C. herbarum* (77). One study also highlights that *A. fumigatus, A. alternata*, and *C. herbarum* are three fungi that are routinely tested positive and most often implicated in paediatric fungal asthma (78).

The other mould that is most studied and frequently included in allergy panels is *P. chrysogenum.* This species is one of the most common indoor moulds, thriving in damp or water-damaged houses, making sensitization to *P. chrysogenum* clinically meaningful in indoor-driven asthma endotypes (79). Pen ch 13 and Pen ch 18 are identified as the main allergens found in *P. chrysogenum,* but they are not species-specific due to cross-reactivity with other moulds (80). The four primary fungal species share a similar pathogenic mechanism, involving secreted proteases. These proteases compromise the integrity of the airway epithelium and activate protease-activated receptor-2 (PAR-2). Despite this shared pathway, each species exhibits distinct features. *A. alternata* is notable for inducing rapid and severe asthma exacerbations via protease-driven IL-33 release (81). *A. fumigatus* introduces complexity with its Asp f 13, which induces airway hyperresponsiveness and degrades the extracellular matrix. Additionally, its other proteases impair antiviral signaling and contribute to airway remodeling (82)*. P. chrysogenum* further induces histamine release, rupturing tight junction barriers, resembling but distinct from *Aspergillus* protease activity (83). *C. herbarum* generally aligns with the protease-PAR-2 model but does not demonstrate the aggressive epithelial or immune disruption seen in *Alternaria* or *Aspergillus* (84).

Apart from moulds*, C. albicans* is primarily a commensal yeast found on mucosal surfaces. It is a very common, if not universal, commensal organism in healthy people. However, it can act as an opportunistic pathogen in immunocompromised individuals and may produce allergenic proteins. Individuals with asthma who use high-dose corticosteroid inhalers may experience oral candidiasis (thrush) due to the suppression of the local immune response in the mouth (85). Furthermore, chronic *Candida* colonization of the oropharynx or bronchial tree may promote persistent low-grade allergic inflammation. Recent studies have discovered that the peptide toxin candidalysin, rather than secreted proteases, is the critical allergen (86). Candidalysin directly binds the platelet glycoprotein Ib*α* (GP1ba) receptor, activating platelets to release the Wnt antagonist Dickkopf-1 (DKK1). DKK1 production drives Th2 and Th17 polarization, leading to eosinophilic inflammation, airway hyper-responsiveness, and mucus over-production—hallmarks of asthmatic pathology. Unlike *Aspergillus* spp., which rely on protease-mediated epithelial damage and IL-33 activation, *C. albicans* induces disease through this candidalysin-platelet-DKK1 axis, highlighting a novel, protease-independent pathway for fungal-associated asthma (87).

*Trichophyton* sp. was the least commonly included allergen in fungal sIgE testing because dermatophytes such as *T. rubrum* and *T. mentagrophytes* are typically skin pathogens. Although the fungus colonizes only skin, nails, and hair, its proteins can be inhaled or reach the upper airway mucosa, allowing the immune system to encounter *Trichophyton* antigens. The primary clinically relevant allergen identified from the dermatophyte *Trichophyton* is the 83-kDa protein Tri t 4. In parallel, *Trichophyton* antigens also induce delayed-type hypersensitivity (DTH) reactions, which are associated with Th17 activation and neutrophilic inflammation (88). Patients with moderate or severe asthma show a markedly higher prevalence of *Trichophyton*-specific IgE (15.8% and 32.4% respectively) compared with controls (7%) or mild asthma (4.9%). The presence of these IgE antibodies is an independent determinant of asthma severity (89). People with poorly controlled asthma and dermatophytosis experience improvement when the skin fungal infection is cleared with fungal therapy, indicating the importance of *Trichophyto*n in perpetuating asthma (90).

### 4.4 Challenges and future directions

Some assays perform well for *Aspergillus* and *Alternaria* but exhibit reduced concordance with less common fungi, such as *Cladosporium, Candida,* and *Trichophyton*. Emerging technologies, particularly microarray-based assays, show great promise, offering improved sensitivity and the ability to profile multiple allergens using minimal sample volumes. However, further validation across diverse populations is needed. This review identifies three key priorities: (1) standardizing cutoff thresholds across assays; (2) including relevant recombinant fungal allergens; and (3) conducting large-scale comparative studies to assess real-world concordance. Addressing these priorities can lead to more accurate diagnoses of fungal asthma, targeted therapies, and enhanced patient outcomes.

## Data Availability

Publicly available datasets were analyzed in this study. This data can be found here: Pubmed publications.
