# Supplementary material for: Concordance between different methods of detecting fungal-specific IgE antibody in asthma
Source: Front Allergy. 2026 Apr 1;7:1788615. doi: 10.3389/falgy.2026.1788615 (PMC13079675; doi:10.3389/falgy.2026.1788615)
Supplement: Supplementary file 1 [file Supplementaryfile1.docx]

# Supplementary materials

Table 1. Descriptive studies of widely used specific IgE antibody detection assays alongside their capabilities in detecting common fungal allergens (n=32)

|  | **Commercial names** | **Manufacturer** | **Country of origin** | **Format** | **Positivity cutoff** | **Fungal allergens detected** | | | | | | **References** |
| --- | --- | --- | --- | --- | --- | --- | --- | --- | --- | --- | --- | --- |
|  |  |  |  |  |  | **Asp** | **Alt** | **Cla** | **Can** | **Tri** | **Pen** |  |
| 1 | ImmunoCAP | Thermo Fisher Scientific | Sweden | EIA^b^ | ≥ 0.35 kU_A_/L | ✔ | ✔ | ✔ | ✔ | ✔ | ✔ | (80), (81), (82), (27) |
| 2 | ImmunoCAP ISAC 112 | Thermo Fisher Scientific | Sweden | Microarray | Semi-quantitative;  ≥ 0.30 ISU-E | ✔ | ✔ | ✔ | ✔ | ✔ | ✔ | (83), (84) |
| 3 | View Allergy | Thermo Fisher Scientific | Sweden | EIA^b^ | ≥ 0.27 Index units (Class 1) | ✔ | ✔ | ✘ | ✔ | ✘ | ✘ | (85) |
| 4 | MeDALL chip^ARG^ | EU Project | France & Spain | Microarray | ≥ 0.30 ISU‑E | ✔ | ✔ | ✔ | ✔ | ✘ | ✔ | (31), (47), (86) |
| 5 | Allergy Line | DST Diagnostics | Germany | EIA^a^ | ≥ 0.35 kU_A_/L | ✔ | ✔ | ✔ | ✘ | ✘ | ✔ | (87) |
| 6 | EUROLINE | EUROIMMUN | Germany | Immunoblot | ≥ 0.35 kU_A_/L | ✔ | ✔ | ✔ | ✔ | ✔ | ✔ | (43), (88) |
| 7 | Allergy Screen | Mediwiss Analytic GmbH | Germany | Immunoblot | Semi-quantitative; Class ≥ 1 | ✔ | ✔ | ✘ | ✘ | ✘ | ✔ | (89), (90) |
| 8 | RIDA qLine Allergy | R‑Biopharm AG | Germany | Immunoblot | ≥ 0.35 kU_A_/L | ✔ | ✔ | ✔ | ✔ | ✘ | ✔ | (91), (92) |
| 9 | Polycheck Allergy | Biocheck GmbH | Germany | Immunoblot | ≥ 0.35 kU_A_/L | ✔ | ✔ | ✔ | ✔ | ✘ | ✔ | (93), (94) |
| 10 | DELFIA | PerkinElmer | Finland | EIA^b^ | cutoff values varies by antigen | ✔ | ✔ | ✔ | ✔ | ✔ | ✔ | (18), (95) |
| 11 | AlaSTAT | Siemens Healthcare Diagnostics | UK | EIA^c^ | ≥ 0.35 kU_A_/L | ✔ | ✔ | ✘ | ✔ | ✘ | ✔ | (26) |
| 12 | IDS sIgE | Immunodiagnostics | UK | EIA^c^ | ≥ 0.1 kU_A_/L | ✔ | ✔ | ✘ | ✔ | ✘ | ✔ | (44) |
|  | **Commercial names** | **Manufacturer** | **Country of origin** | **Format** | **Positivity cutoff** | **Fungal allergens detected** | | | | | | **References** |
|  |  |  |  |  |  | **Asp** | **Alt** | **Cla** | **Can** | **Tri** | **Pen** |  |
| 13 | IMMULITE 2000 | Siemens Healthcare Diagnostics | UK | EIA^c^ | ≥ 0.10 kU_A_/L | ✔ | ✔ | ✔ | ✔ | ✘ | ✔ | (96), (97) |
| 14 | ALEX-2 | MacroArray Diagnostics | Austria | EIA^a^ | ≥ 0.30 kU_A_/L | ✔ | ✔ | ✔ | ✔ | ✔ | ✔ | (84), (98) |
| 15 | MosaiQ® AllergyPlex™ COMBO | AliveDx | Switzerland | Microarray | Not publicly disclosed; | ✔ | ✔ | ✔ | ✘ | ✘ | ✘ | (99) |
| 16 | HYTEC 288 | HYCOR Biomedical | USA | EIA^a^ | ≥ 0.35 kU_A_/L | ✔ | ✔ | ✘ | ✔ | ✘ | ✘ | (100), (101) |
| 17 | IgE inhalant allergy test | Mosaic Diagnostics | USA | EIA^a^ | ≥ 0.35 kU_A_/L | ✘ | ✔ | ✘ | ✘ | ✘ | ✘ | (102) |
| 18 | NOVEOS | HYCOR Biomedical | USA | EIA^c^ | ≥ 0.35 kU_A_/L | ✔ | ✔ | ✘ | ✘ | ✘ | ✘ | (103), (104) |
| 19 | OPTIGEN | Minaris Medical America | USA | EIA^c^ | Class 1 with ≥ 501 LMC | ✔ | ✔ | ✘ | ✘ | ✘ | ✘ | (105), (92) |
| 20 | AllergoELISA | AlkorBio | Russia | EIA^a^ | ≥ 0.35 kU_A_/L | ✔ | ✘ | ✘ | ✘ | ✘ | ✘ | (106), (107) |
| 21 | Allergy Advance Panel | Redcliffe Labs | India | EIA^a^ | ≥ 0.35 kU_A_/L | ✔ | ✔ | ✔ | ✔ | ✔ | ✔ | (108) |
| 22 | Accuris B+ve Allergy Panel | Sterling Accuris Diagnostics | India | EIA^a^ | ≥ 0.35 kU_A_/L | ✔ | ✔ | ✘ | ✔ | ✘ | ✔ | (109) |
| 23 | BioIC | Agnitio Science Technology | China | EIA^c^ | ≥ 0.35 kU_A_/L | ✔ | ✔ | ✔ | ✔ | ✘ | ✔ | (44), (110) |
| 24 | BioCLIA® Allergy | HOB Biotech Group Corp | China | EIA^b^ | ≥ 0.35 kU_A_/L | ✔ | ✔ | ✘ | ✘ | ✘ | ✘ | (111), (112) |
| 25 | Ouboke | HOB Biotech Group Corp | China | EIA^a^ | ≥ 0.35 kU_A_/L | ✔ | ✔ | ✔ | ✔ | ✘ | ✔ | (44), (113) |
| 26 | Single/Multivent Allergen series | Hangzhou Zheda Dixun Biological  Engineering | China | Immunoblot | ≥ 0.35 kU_A_/L | ✔ | ✔ | ✔ | ✘ | ✘ | ✔ | (114) |
|  |  |  |  |  |  |  |  |  |  |  |  |  |
|  | **Commercial names** | **Manufacturer** | **Country of origin** | **Format** | **Positivity cutoff** | **Fungal allergens detected** | | | | | | **References** |
|  |  |  |  |  |  | **Asp** | **Alt** | **Cla** | **Can** | **Tri** | **Pen** |  |
| 27 | IgE Antibody REAST Kit | HOB Biotech Group Corp., Ltd. | China | REAST | ≥ 0.35 kU_A_/L | ✘ | ✔ | ✘ | ✘ | ✘ | ✘ | (115), (113) |
| 28 | Oriton IgE kit | Nippon Chemipha Corporation | Japan | EIA^a^ | ≥ 0.35 kU_A_/L | ✔ | ✘ | ✘ | ✔ | ✘ | ✘ | (28) |
| 29 | GOLD chip^ARG^ | Korean project | South Korea | Microarray | ≥ 0.35 kU_A_/L | ✔ | ✔ | ✔ | ✘ | ✘ | ✘ | (116) |
| 30 | MAST-CLA | Hitachi Chemical Diagnostics | South Korea | EIA^c^ | Class ≥ 1 with ≥ 1.39 LMC | ✔ | ✔ | ✘ | ✘ | ✘ | ✘ | (105), (92) |
| 31 | PROTIA Allergy-Q | ProteomeTech Inc. | South Korea | EIA^a^ | ≥ 0.35 kU_A_/L | ✔ | ✔ | ✘ | ✔ | ✔ | ✔ | (30) |
| 32 | AdvanSure | LG Chem | South Korea | EIA^a^ | ≥ 0.35 kU_A_/L | ✔ | ✔ | ✘ | ✔ | ✘ | ✔ | (117), (116) |
|  | **Positive detections** | | | | | **30/32**  **30/32** | **30/32**  **30/32** | **16/32**  **16/32** | **20/32**  **21/32** | **7/32**  **7/32** | **20/32**  **21/32** |  |

Table 2. Comparative studies of the diagnostic performance of different systems for detecting IgE asthma-related allergens (*n=*13)

| **Authors, years** | **Country** | **Assays** | **Study size** | **Allergen types** | **Comparison of metrics outcomes** |
| --- | --- | --- | --- | --- | --- |
| Kim, 2011  (105) | South Korea | **MAST CLA**  *VS*  **OPTIGEN** | **Study group:**  A total of 944 patients who had symptoms of rhinitis at Kon-Kun University Hospital were sorted into 4 different groups   - Group 1, *n=*251, tested with MAST-CLA and skin prick test (SPT) - Group 2*, n=*319, tested with OPTIGEN and SPT - Group 3, *n*=104, tested with MAST-CLA and ImmunoCAP - Group 4, *n*=270, tested with OPTIGEN and ImmunoCAP   **Control group:**  None. However, it did compare the two diagnostic kits with ImmunoCAP and SPT. | *n=* 8 allergens  (fungal allergens included are *Alternaria sp.* and *Aspergillus sp.*) | - ICCs in Group 1 and Group 3 were above 0.5, only for *Dermatophagoides* sp. - ICCs in Group 2 were above 0.5, only for *Dermatophagoides* sp. and cat allergens - ICCs in Group 4 were above 0.5, except for the cockroach   The overall agreement with ImmunoCAP and SPT showed that the OPTIGEN test performed better than the MAST CLA system |
| Hyun Lee, 2012  (117) | South Korea | **AdvanSure**  *VS*  **ImmunoCAP** | **Study group:**  A total of 199 Korean allergy patients, ranging in age from 2 to 74 years, with a median age of 13 years. Subjects have an allergic history, including   - asthma (39.7%), - allergic rhinitis (54.8%), - atopic dermatitis (36.2%), and food allergies (21.6%)   **Control group:**  None. | *n=* 13 allergens  (fungal allergen included is *Alternaria sp.)* | - ICCs was above 0.5, except *B. germanica, Alternaria,* and mugwort sIgEs. - The inter-method concordance was moderate to substantial (κ=0.528–0.778*, P*<0.001) - Total agreement was 76.0% to 93.1% (55.0–88.9% for positive and 73.7–95.4% for negative agreement). |
| Han, 2013  (92) | South Korea | **RIDA qLine Allergy**  *VS*  **MAST Optigen**  *VS*  **Polycheck Allergy** | **Study group:**  A total of 40 sera tested positive with the RIDA Allergy Screen at Seoul National University Hospital,  **Control group:**  None. However, it did compare the three diagnostic kits with ImmunoCAP | *n=* 40 allergens  (fungal allergens included are *Aspergillus sp., Cladosporium sp., Alternaria sp.,* *Penicillium sp*., and *C. albicans*) | - Total agreement between RIDA and MAST Optigen system was 87.6% for inhalant allergen and 91.3% for food allergen - Total agreement between RIDA and the Polycheck system was 89.9% for inhalant allergens and 88.3% for food allergens - Total agreement between MAST and the Polycheck system was 86.8% for inhalant allergens and 90.6% for food allergens - Compared with ImmunoCAP, MAST Optigen showed better agreement than the other two assays |
| Lee,  2014  (100) | South Korea | **ImmunoCAP**  *VS*  **HYTEC 288** | **Study group:**  A total of 65 patients with suspected allergic diseases underwent *in vitro* allergy testing at Yangsan Pusan University Hospital. The average age of patients ranged from 0 to 74 years.  **Control group:**  Each serum sample was tested for specific allergens, including *D. pteronyssinus* (d1) and *D. farinae* (d2). | *n=* 21 allergens  (fungal allergens included are *A. alternata* and *A. fumigatus)* | - Overall agreement was 90% to all allergens, with *A. alternata* showing 88.9% concordance and *A. fumigatus* showing complete agreement |
| Cho, 2014  (118) | South Korea | **Skin Prick Test (SPT)**  *VS*  **AdvanSure**  *VS*  **ImmunoCAP** | **Study group:**  A total of 100 patients with chronic rhinitis were recruited from the outpatient otolaryngology clinic at Konkuk University Hospital in Seoul, Korea. The patients’ average age is 42.3 years.  **Control group:**  40 healthy adult patient volunteers with the average age of 44.5 years | *n=* 10 allergens  (fungal allergens included are *A. alternata)* | - Total agreement between ImmunoCAP and AdvanSure was 84-99%, with inter-method concordance was fair to substantial (κ=0.310–0.884*, P*<0.001) - Total agreement between ImmunoCAP and SPT was 87-97%, with inter-method concordance was fair to substantial (κ=0.394–0.816*, P*<0.001) - Total agreement between AdvanSure and SPT was 88-96%, with inter-method concordance was fair to substantial (κ=0.335–0.789*, P*<0.001) |
| Lee, 2015  (30) | South Korea | **PROTIA^TM^ Allergy-Q®**  *VS*  **ImmunoCAP®** | **Study group:**  260 Korean patients with allergies, aged 1 to 75 years, were clinically diagnosed with IgE-related allergic diseases, including   - asthma (26.5%), - allergic rhinitis (42.3%), - atopic dermatitis (67.7%), - and food allergy (18.15%).   **Control group:**  None. | *n=* 19 allergens  (fungal allergens included are *A. alternata,* *C. albicans*, and *T. rubrum*) | - ICCs was above 0.5, except *Alternaria,* and mugwort - The inter-method concordance was moderate to substantial (κ=0.713-0.898, *P*<0.001) - Total agreement was 86.63%-96.63% between the two systems |
| Hwang, 2016  (91) | South Korea | **ImmunoCAP Phadiatop**  *VS*  **RIDA qLine Allergy** | **Study group:**  A total of 430 consecutive patient samples exhibiting allergic symptoms at Kosin University Gospel Hospital in South Korea.  **Control group:**  None. | *n=* 14 allergens  (fungal allergens included are *P. chrysogenum* and *A. alternata*) | - The inter-method concordance was substantial (κ=0.614, *P*<.001) - Total agreement was 80.7% (42.6% positive agreement and 38.1% negative agreement) - The RIDA qLine Allergy test has a higher false-positive rate for some allergens than the Phadiatop test |
| Knight, 2018  (101) | USA | **Skin Prick Test (SPT)**  *VS*  **HYTEC™288** | **Study group:**  A total of 232 serum samples from patients with a request for SPT, obtained from the National Jewish Health (NJH) Biobank.  **Control group:**  None. | *n=* 13 allergens  (fungal allergens included are *A. fumigatus* and *A. alternata* | - The overall agreement between SPT and HYTEC™288 sIgE was >70% for every allergen examined. - Sensitivity varied between 25% and 95%, whereas specificity was greater for all allergens (78–97%). - The NPV exceeded 85% for every allergen evaluated, whereas the PPV showed more variability, fluctuating between 22% and 88%. |
| Park, 2018  (97) | South Korea | **Immulite 2000 Allergy System**  *VS*  **ImmunoCAP 250 system** | **Study group:**  A total of 209 Korean patients with allergies, aged 6 to 80 years, were diagnosed with   - allergic rhinitis (61.2%), - asthma (40.2%), - atopic dermatitis (31.6%), food allergy (18.2%), and - chronic urticaria (13.9%).   **Control group:**  None. | *n=* 14 allergens  (fungal allergens included are *A. alternata*) | - ICCs average was above 0.5 - The inter-method concordance was moderate to substantial, (κ=0.413-0.923, *P*<0.001) - The agreement ratios for positivity and negativity ranged from 75% for wheat and shrimp to 96% for *Alternaria*. |
| Pajor, 2020  (110) | Poland | **BioIC**  *VS*  **ImmunoCAP** | **Study group:**  A total of 20 participants (aged 1–34 years; 11 female and 9 male) underwent routine sIgE assessment.  **Control group:**  None. | *n=* 33 allergens  (fungal allergens included are *A. alternata*) | - ICCs was 1 (p < 0.05) for all tested allergens. - The inter-method concordance was perfect, (κ=1.0, *P*<0.001) - Total agreement was 100% for positivity and negativity |
| Skevaki, 2021  (43) | Germany | **ImmunoCAP**  *VS*  **ImmunoCAP ISAC 112**  *VS*  **Euroline™** | **Study group:**  Serum samples from 82 children, with an average age of 4 years, were diagnosed with asthma or wheeze.  **Control group:**  24 children without recurrent asthma or wheeze | *n=* 22 allergens  (fungal allergens included are *Cladosporium sp*.) | - All three diagnostic kits showed good sensitivity and specificity, with AUC value consistently >0.7 - The levels of NPV were high (over 82% to over 89%), whereas PPV showed significant variation (ranging from 0% to 100%). |
| Yoshikawa, 2021  (28) | Japan | **Oriton IgE**  *VS*  **ImmunoCAP**  *VS*  **AlaSTAT** | **Study group:**  A total of 255 anonymized sample patients underwent sIgE antibody testing.  **Control group:**  None. | *n=* 6 allergens  (fungal allergens included are *Candida sp.)* | - Oriton IgE test using 50μL matched the ImmunoCAP test 93.7% of the time. While the 89.8% match rate is with the Alastat test - Oriton IgE using the 30μL method, increase the concordance rate to 95.3%, with ImmunoCAP and 90.6% with Alastat test - *Candida sp.* showed a lower concordance, with 0.923% for ImmunoCAP and 0.920% for Alastat |
| Galvan, 2024  (119) | Peru | **Peruvian Immunoblot**  *VS*  **Skin Prick Test (SPT)**  *VS*  **ALEX-2** | **Study group:**  35 patients, including children over 3 years old up to adults with a median age of 9 years and an interquartile range of 6–30 years. Patients suspected of having allergic rhinitis.  **Control group:**  None. | *n=* 29 allergens  (fungal allergens included are *Alternaria sp.* and *Cladosporium sp.)* | - The concordance between ALEX-2 and SPT was fair to nearly perfect,   (κ=−0.04-0.999)   - The concordance between ALEX-2 and Peruvian Immunoblot was fair to substantial (κ=−0.029-0.717) - The concordance between SPT and Peruvian Immunoblot was fair to moderate (κ=−0.290-0.598) - ALEX-2 demonstrated higher sensitization rates for specific allergens and had greater agreement with SPT |

ICCs: Intraclass coefficient correlation, values above 0.5 = moderate to substantial reliability measurements

**Table 2**. *(continued)*

**Table 2**. *(continued)*

Cohen’s kappa values (κ)= almost perfect (0.8–1.0), substantial (0.6–0.8), moderate (0.4–0.6), fair (0.2–0.4), and poor (<0.2)

PPV: Positive Predictive Value

NPV: Negative Predictive Value

Table 3. Comparative studies on diagnostic systems for detecting fungal-specific IgE only (*n=*3)

| **Authors, years** | **Country** | **Assays** | **Fungal allergens** | **Study size** | **Comparison of metrics outcomes** |
| --- | --- | --- | --- | --- | --- |
| Kespohl, 2016  (120) | Germany | **Skin Prick Test (SPT)**  from four different manufacturers in Germany (Aller-Gopharma, ALK-Abello, HAL, and Lofarma)  *VS*  **ImmunoCAP** | - *A. fumigatus* (Asp f) - *C. herbarum* (Cla h) - *P. chrysogenum*   (Pen ch)   - *A. alternata* (Alt a) - *A. versicolor* (Asp v) | **Study group:**  A total of 168 participants were gathered from 13 allergy clinics and practices. Patients had a history or self-reported concerns and/or diagnoses related to mould allergy, exposure, and/or allergic symptoms caused by mould. The average age of the participants was 44 years, with 51% of them being male.  **Control group:**  None. | - Mould sensitization was identified more often through the SPT (90 out of 168) compared to the sIgE tests (56 out of 168) - Double SPT positives reached ≥80% only for environmental allergens and specific mould solutions like Asp f and Alt a, while other mould solutions had concordances below 80% - Concordance rates vary by mould species and manufacturers, with *A. alternata* (Alt a) demonstrating the highest rate at 69% of subjects testing positive |
| Kuwabara, 2018  (85) | Japan | **ImmunoCAP**  *VS*  **View Allergy**  *VS*  **MAST IV** | - *A. fumigatus* | **Study group:**  A total of 57 patients were diagnosed with bronchial asthma, with a subset also diagnosed with ABPA. Patients were recruited at Fujita Health University Teaching Hospital  **Control group:**  None. | - The concordance rate between ImmunoCAP and View Allergy was 98.2%, (positive concordance ratio of 100% and a negative concordance ratio of 96.9%) - The concordance rate between ImmunoCAP and MAST IV was 77.2% (a positive concordance ratio of 45.8% and a negative concordance ratio of 100%) - View Allergy provides more consistent results with the gold standard, ImmunoCAP than MAST |
| Huang, 2025  (44) | China | **ImmunoCAP**  *VS*  **BioIC**  *VS*  **EUROLine**  *VS*  **Ouboke** | - Fungal mixtures - *(P. chrysogenum,*   - *C. herbarum,*   - *C. albicans,*   - *A. alternata, S. rostrata),* and *A. fumigatus* | **Study group:**  A total of 99 patients with ABPA were divided into two groups based on the tIgE level: ≥ 1000 kUA/L as the Confirmed group and 100–1000 kUA/L as the Probable group. The average age of the participants was 33 (23-50) years, 56 were male and 43 were female.  **Control group:**  30 patients with a negative SPT for *A. fumigatus* | - **ImmunoCAP system;** - Sensitivity on fungal mixture was 90.9% and the specificity was 90.0% - Sensitivity on *A. fumigatus* was 88.9% and the specificity was 100%. - **BioIC system;** - Sensitivity on fungal mixture was 38.4% and the specificity was 96.7% - Sensitivity on *A. fumigatus* was 26.3% and the specificity was 96.7% - **EUROLine system;** - Sensitivity on fungal mixture was 44.44% and the specificity was 100% - **Ouboke system;** - Sensitivity on fungal mixture was 52.5% and the specificity was 90.0% |

**A. fumigatus* remains the primary target, present in all fungal sIgE comparative studies

**Table 3**. *(continued)*

Table 4. Summary of the most common fungal allergens and their association with asthma endotypes

| **Fungi** | ***A. fumigatus*** | ***A. alternata*** | ***C. herbarum*** | ***C. albicans*** | ***T. rubrum*** | ***P. chrysogenum*** |
| --- | --- | --- | --- | --- | --- | --- |
| **Primary source of exposure** | Airborne spores | Airborne spores | Airborne spores | Mucosal colonization | Skin and nail infections | Airborne spores |
| **Major allergens** | Asp f 1, f 2, f 3,  f 4, f 6 | Alt a 1 | Cla h 8 | Candidalysin | Tri t 4 | Pen ch 13,  Pen ch 18 |
| **Specific IgE cutoff values** | A level of 0.35 kU_A_/L is frequently used, but elevated levels (0.70 kU_A_/L) are more indicative of a disease rather than mere incidental sensitization. | Even low-level positivity (≥0.35 kUA/L) has been linked to the risk of severe exacerbations | Most studies use the standard ≥0.35 kUA/L | Most studies use the standard ≥0.35 kUA/L | Most studies use the standard ≥0.35 kUA/L | Most studies use the standard ≥0.35 kUA/L |
| **Associated asthma endotypes** | ABPA, ABPM, SAFS, paediatric asthma | SAFS, thunderstorm asthma, paediatric asthma | SAFS, seasonal asthma, paediatric asthma | SAFS, steroid-dependent asthma | SAFS, systemic sensitization | SAFS, indoor mould-driven asthma |
| **References** | (121), (122) | (64), (60) | (66), (67) | (74), (75) | (77), (78) | (68), (69) |

ABPA: allergic bronchopulmonary aspergillosis

ABPM: allergic bronchopulmonary mycosis

SAFS: severe asthma with fungal sensitisation

**REFERENCES**

18. Barrera C, Schwarz C, Delhaes L, Le Gal S, Ramel S, Gangneux JP, Guitard J, Hoffmann C, Bellanger AP, Bouchara JP, Millon L. Detection of Specific IgE against Molds Involved in Allergic Bronchopulmonary Mycoses in Patients with Cystic Fibrosis. Mycopathologia. 2024;189(4):68.

26. NIAID-Sponsored Expert Panel; Boyce JA, Assa'ad A, Burks AW, Jones SM, Sampson HA, Wood RA, Plaut M, Cooper SF, Fenton MJ, Arshad SH, Bahna SL, Beck LA, Byrd-Bredbenner C, Camargo CA Jr, Eichenfield L, Furuta GT, Hanifin JM, Jones C, Kraft M, Levy BD, Lieberman P, Luccioli S, McCall KM, Schneider LC, Simon RA, Simons FE, Teach SJ, Yawn BP, Schwaninger JM. Guidelines for the diagnosis and management of food allergy in the United States: report of the NIAID-sponsored expert panel. . J Allergy Clin Immunol 2010;126(6 Suppl):S1–S58. .

28. Yoshikawa Y, Nakashita T, Motojima S, Otsuka Y. Allergy-specificIgE Measurement Reagent Oriton IgE and ImmunoCap and Alastair Correlation. Allergy. 2022;71(2).

30. Lee JH, Park HJ, Park KH, Jeong KY, Park JW. Performance of the PROTIATM Allergy-Q® System in the Detection of Allergen-specific IgE: A Comparison With the ImmunoCAP® System. Allergy Asthma Immunol Res. 2015;7(6):565-572.

31. Lupinek C, Wollmann E, Baar A, Banerjee S, Breiteneder H, Broecker BM, et al. Advances in allergen-microarray technology for diagnosis and monitoring of allergy: the MeDALL allergen-chip. Methods. 2014;66(1):106-19.

43. Skevaki C, Tafo P, Eiringhaus K, Timmesfeld N, Weckmann M, Happle C, et al. Allergen extract- and component-based diagnostics in children of the ALLIANCE asthma cohort. Clin Exp Allergy. 2021;51(10):1331-45.

44. Huang Z, Li W, Chen H, Sun B. Evaluation of the clinical performance of four fungus-specific immunoglobulin E detection systems in patients with Aspergillus allergy. Asian Pacific Journal of Allergy and Immunology. 2025;43(1).

47. Skrindo I, Lupinek C, Valenta R, Hovland V, Pahr S, Baar A, et al. The use of the MeDALL-chip to assess IgE sensitization: a new diagnostic tool for allergic disease? Pediatr Allergy Immunol. 2015;26(3):239-46.

60. Knutsen AP, Bush R, Demain JD, Denning DW, Dixit A, Fairs A, Greenberger PA, Kita H, Kariuki B, Kurup VP, Moss RB, Niven RM, Pashley CH, Slavin RG, Vijay H, Wardlaw AJ. Fungi and allergic lower respiratory tract diseases. Journal of Allergy and Clinical Immunology. 2012;129(2):280-91.

64. Abel-Fernández E, Martínez MJ, Galán T, Pineda F. Going over Fungal Allergy: Alternaria alternata and Its Allergens. J Fungi (Basel). 2023;18;9(5):582.

66. Solutions DCFaTH. m2 Cladosporium herbarum: Thermo Fisher Scientific; 2021 [cited 2025 22 August]. Available from: <https://www.thermofisher.com/phadia/gb/en/resources/allergen-encyclopedia/m2.html>.

67. Bush A. Kids, Difficult Asthma and Fungus. Journal of Fungi. 2020;6(55).

68. Gent JF, Kezik JM, Hill ME, Tsai E, Li DW, Leaderer BP. Household mold and dust allergens: Exposure, sensitization and childhood asthma morbidity. Environ Res 2012(118):86-93.

69. Solutions DCFaTH. m1 Penicillium chrysogenum 2021 [cited 2025 August 22nd]. Available from: <https://www.thermofisher.com/phadia/wo/en/resources/allergen->

74. Dekhuijzen PNR, Batsiou M, Bjermer L, Bosnic-Anticevich S, Chrystyn H, Papi A, Rodríguez-Roisin R, Fletcher M, Wood L, Cifra A, Soriano JB, Price DB. Incidence of oral thrush in patients with COPD prescribed inhaled corticosteroids: Effect of drug, dose, and device. Respiratory Medicine. 2016;120:54-63.

75. Wu Y, Zeng Z, Guo Y, Song L, Weatherhead JE, Huang X, Zeng Y, Bimler L, Chang CY, Knight JM, Valladolid C, Sun H, Cruz MA, Hube B, Naglik JR, Luong AU, Kheradmand F, Corry DB. Candida albicans elicits protective allergic responses via platelet mediated T helper 2 and T helper 17 cell polarization. Immunity. 2021;54(11):2595-610. encyclopedia/m1.html#:~:text=was%20found%20to%20be%201.5,synthesized%20only%20under%20specific%20circumstances.

77. Matsuoka H, Niimi A, Matsumoto H, Ueda T, Takemura M, Yamaguchi M, Jinnai M, Otsuka K, Oguma T, Takeda T, Ito I, Chin K, Amitani R, Mishima M. Specific IgE Response to Trichophyton and Asthma Severity. CHEST. 2009;135(14):898-903.

78. Platts-Mills TAE, Woodfolk JA. Trichophyton Asthma. CHEST. 2009;135(4):887-8.

80. Schoos AM, Hansen SM, Skov FR, Stokholm J, Bønnelykke K, Bisgaard H, Chawes BL. Allergen Specificity in Specific IgE Cutoff. JAMA Pediatr 2020;18;174(10):993–995.

81. Scientific TF. ImmunoCAP™ Specific IgE 2019 [29 July 2025]. Available from: https://dfu.phadia.com/Data/Pdf/5dae9e2489c23208b8036206.pdf.

82. Kwizera R, Bongomin F, Olum R, Meya DB, Worodria W, Bwanga F, Fowler SJ, Gore R, Denning DW, Kirenga BJ. Fungal asthma among Ugandan adult asthmatics. Medical Mycology. 2021;59, 923–933.

83. Martínez-Aranguren R, Lizaso MT, Goikoetxea MJ, García BE, Cabrera-Freitag P, Trellez O, Sanz ML. Is the Determination of Specific IgE against Components Using ISAC 112 a Reproducible Technique? PLoS One. 2014;9(2):e88394.

84. Nosslinger H, Mair E, Oostingh GJ, Ahlgrimm-Siess V, Ringauf A, Lang R. Multiplex Assays in Allergy Diagnosis: Allergy Explorer 2 versus ImmunoCAP ISAC E112i. Diagnostics (Basel). 2024;14(10).

85. Kuwabara K, Yokoi T, Yoshida T, Shiga M, Hirose M, Kondo R, Matsunaga K, Nakamura M, Horiguchi T. Comparison between ImmunoCAP and multiple antigen simultaneous tests for measuring Aspergillus-specific Immunoglobulin E levels in Aspergillus-sensitized patients. Fujita Medical Journal. 2018;4(3).

86. GALEN. About Project MeDALL: Global Allergy and Asthma Excellence Network i; 2010 [cited 2025 12 August]. Available from: <https://global-allergy.network/portfolio/project-medall/>.

87. DST. Instruction For Use (IFU) Allergy Line. In: GmbH DDST, editor. 2022.

88. Wongpiyabovorn J, Suratannon N, Boonmee S, Chatchatee P. Comparison of specific IgE detection by immunoblotting and fluorescence enzyme assay with in vivo skin prick test. Asian Pac J Allergy Immunol. 2018;36(3):159-65.

89. MEDIWISS. Allergy Screen. In: GmbH MA, editor. Diagnostic of allergy diseases. Germany2019.

90. Koch L, Aberer W. Comparability and quality of IgE-based in vitro allergy diagnosis: 25 years of external quality assessment. Wien Klin Wochenschr. 2014;126(19-20):634-41.

91. Hwang H, Kwon J, Kim JY, Lee HH, Oh CE, Choi GS. The RIDA Allergy Screen Versus the Phadiatop Test in 430 Consecutive Patient Specimens. Lab Med. 2016;47(1):20-9.

92. Han M, Shin S, Park H, Park KU, Park MH, Song EY. Comparison of three multiple allergen simultaneous tests: RIDA allergy screen, MAST optigen, and polycheck allergy. Biomed Res Int. 2013;2013:340513.

93. Polycheck. Polycheck Product Catalog : Serum Diagnostics. In: GmbH B, editor. Germany2022.

94. Jang WR, Nahm CH, Kim JH, Lim DH, Jang TY, Moon YS, Kim JJ. Allergen specific IgE measurement with Polycheck Allergy: comparison of three multiple allergen simultaneous tests. Korean J Lab Med. 2009;5:465-72.

95. Barrera C, Richaud-Thiriez B, Rocchi S, Rognon B, Roussel S, Grenouillet F, Laboissière A, Dalphin JC, Reboux G, Millon L. New Commercially Available IgG Kits and Time-Resolved Fluorometric IgE Assay for Diagnosis of Allergic Bronchopulmonary Aspergillosis in Patients with Cystic Fibrosis. Clinical and Vaccine Immunology. 2016;23(3):196–203.

96. Yang J, Lee H, Choi AR, Park KH, Ryu JH, Oh EJ. Comparison of allergen-specific IgE levels between Immulite 2000 and ImmunoCAP systems against six inhalant allergens and ten food allergens. Scand J Clin Lab Invest. 2018;78(7-8):606-12.

97. Park KH, Lee J, Sim DW, Lee SC. Comparison of Singleplex Specific IgE Detection Immunoassays: ImmunoCAP Phadia 250 and Immulite 2000 3gAllergy. Ann Lab Med. 2018;38(1):23-31.

98. Quan PL, Sabaté-Brescó S-BM, D'Amelio CM, Pascal M, García BE, Gastaminza G, Blanca-López N, Alvarado MI, Fernández J, Moya C, Bartra J, Ferrer M, Goikoetxea MJ. Validation of a commercial allergen microarray platform for specific immunoglobulin E detection of respiratory and plant food allergens. Ann Allergy Asthma Immunol. 2022;28(3):283-290.

99. AliveDx. MosaiQ AllergyPlex® COMBO Swiss: AliveDx; 2024 [8 August 2025]. Available from: https://alivedx.com/mosaiqs-allergyplex-assay/.

100. Lee SH, Lee SM, Kim HH, Chang CL, Lee EY. Performance Comparison of ImmunoCAP and HYTEC 288 in the Quantitative Tests of Allergen-specific IgE. Laboratory Medicine Online. 2014;4(1).

101. Knight V, Wolf ML, Trikha A, Curran-Everett D, Hiserote M, Harbeck RJ. A comparison of specific IgE and skin prick test results to common environmental allergens using the HYTEC™ 288. 2018.

102. Walk-In-Lab. IgE Inhalant Allergy Test, Serum - Mosaic Diagnostics Test Kit 2025 [8 August 2025]. Available from: <https://www.walkinlab.com/products/view/ige-inhalant-allergy-test-serum-mosaic-diagnostics-test-kit>.

103. Potapova E BD, Villella V, Meneguzzi G, Scala E, Sfika I, Tripodi S, Panetta V, Dramburg S, Skevaki C, Matricardi PM. Validation study of a new chemiluminescent singleplex IgE assay in a set of Italian allergic rhinitis patients. Clinical and Experimental Allergy. 2020.

104. PharmD BT-E. NOVEOS and ImmunoCAP Have Similar Performances for Diagnosing Food Allergies. Journal of Allergy and Clinical Immunology. 2024;12(6).

105. Kim JK, Yoon YM, Jang WJ, Choi YJ, Hong SC, Cho JH. Comparison study between MAST CLA and OPTIGEN. Am J Rhinol Allergy. 2011;25(4):e156-9.

106. Ya.I. Kozlova NYV, E.V. Frolova, A.E. Uchevatkina, L.V. Filippova, N..V. Vasilyeva. The spectrum of sensitization to aeroallergens as one of the risk factors for uncontrolled severe bronchial asthma. Russian Journal of Allergology. 2023;20.

107. Alkordesign. Allergy Diagnostics: Accurate, Fast, and Available: ALKORBIO; 2010 [11 August 2025]. Available from: https://alkorbiogroup.ru/en/vse_novosti/news_alkorbio/allergy_diagnostics_accurate_fast_and_available.

108. Labs R. Allergy Panel Advanced- Inhalation/Asthma/Rhinitis (33 Allergen). IgE test: Redcliffe Labs; 2025.

109. Accuris. Accuris B+ve Allergy Panel (Food, Drug, Inhalant) in Una: Sterling Accuris; 2023 [11 August 2025]. Available from: <https://sterlingaccuris.com/test/una/accuris-bve-allergy-panel-food,-drug,-inhalant?branch_slug=sterling-accuris-pathology-una#Overview>.

110. Szymczak-Pajor I, Pawliczak R. Comparison of an automated microfluidic immunoassay technology (BioIC, lab-on-chips) and ImmunoCAP assay. Lab-on-chips as a tool for specific IgE (sIgE) detection. Postepy Dermatol Alergol. 2020;37(1):56-60.

111. HOB. Products HOB Biotech Group: HOB Biotech Group; 2025 [cited 2025 11 August]. Available from: <https://www.bioarrow.com/en/page/hob>.

112. HOB. Allergy: HOB Biotech Group; 2025 [cited 2025 11 August]. Available from: http://en.hob-biotech.com/productdetail-1127.html.

113. Li Y , Li L. A multicenter analysis on the changes of sIgE in China during the early period of COVID‐19 pandemic. Immun Inflamm Dis. 2023;7;11(11).

114. ZDBiogene. Allergen-specific antibody IgE test series: Hangzhou Zheda Dixun Biological Gene Engineering Co.; 2019 [cited 2025 11 August]. Available from: <https://www.zdbiogene.com/M/p2/show/id/48.html>.

115. Zhao J, Liang S, Zhou B, Li M, Li L. A Comprehensive Analysis of Immunoglobulin E Levels, Allergen-Specific Sensitivities, and Clinical Manifestations in Allergic Dermatological Conditions: A Multicenter Retrospective Study in China. Clin Cosmet Investig Dermatol. 2024;1;17:499–512.

116. Jeon H, Jung JH, Kim Y, Kwon Y, Kim ST. Allergen Microarrays for In Vitro Diagnostics of Allergies: Comparison with ImmunoCAP and AdvanSure. Ann Lab Med 2018;38(4):338–347.

117. Lee JH, Park KH, Kim HS, Kim KW, Sohn MH, Kim CH, Lee JS, Hong CS, Park JW. Specific IgE measurement using AdvanSure® system: Comparison of detection performance with ImmunoCAP® system in Korean allergy patients. Clinica Chimica Acta. 2012;413:914-9.

118. Cho JH, Suh JD, Kim JK, Hong SC, Park IH, Lee HM. Correlation between skin-prick testing, individual specific IgE tests, and a multiallergen IgE assay for allergy detection in patients with chronic rhinitis. Am J Rhinol Allergy. 2014;28(5):388-91.

119. Galván C, Totesaut M, Muñoz E, Durán R, La Rosa C, Calderón O. Agreement of Peru-Based Allergy Tests for Respiratory Allergens in Allergic Rhinitis Patients. 2024.

120. Kespohl S, Maryska S, Bünger J, Hagemeyer O, Jakob T, Joest M, et al. How to diagnose mould allergy? Comparison of skin prick tests with specific IgE results. 2016.

121. Hamada Y, Fukutomi Y, Nakatani E, Saito A, Watai K, Kamide Y, Sekiya K, Nagai T, Harada K, Shiraishi Y, Oguma T, Asano K, Taniguchi M. Optimal Aspergillus fumigatus and Asp f 1 serum IgG cut-offs for the diagnosis of allergic bronchopulmonary aspergillosis. Allergology International. 2021;70(1):74-80.

122. Muthu V, Singh P, Choudhary H, Sehgal IS, Dhooria S, Prasad KT, Aggarwal AN, Garg M, Chakrabarti A, Agarwal R. Diagnostic Cutoffs and Clinical Utility of Recombinant Aspergillus fumigatus Antigens in the Diagnosis of Allergic Bronchopulmonary Aspergillosis. J Allergy Clin Immunol Pract. 2020;8:579-87.
